# Single Nucleotide Variants (SNVs) of Angiotensin-Converting Enzymes (ACE1 and ACE2): A Plausible Explanation for the Global Variation in COVID-19 Prevalence

**DOI:** 10.1155/2023/9668008

**Published:** 2023-04-03

**Authors:** Saad Mahjub Atiku, Dennis Kasozi, Katrina Campbell

**Affiliations:** ^1^Department of Medical Laboratory Sciences, Department of Nursing and Midwifery, Faculty of Health Sciences, Muni University, P. O. Box 725 Arua, Uganda; ^2^Department of Biochemistry, Habib Medical School Faculty of Health Sciences, Islamic University in Uganda, P. O. Box 7689 Kampala, Uganda; ^3^Department of Biochemistry and Sports Science, College of Natural Sciences, Makerere University Kampala, P. O. Box 7062 Kampala, Uganda; ^4^Institute for Global Food Security, School of Biological Sciences, OG.076 / O2.037, 19 Chlorine Gardens, Queen's University Belfast, BT9 5DL, UK

## Abstract

**Background:**

Although it is common knowledge that the coronavirus disease of 2019 (COVID-19) and other viral infections have an uneven impact globally, the reasons for this are still indistinct. The absence of equivalent capacities worldwide in screening, testing, and reporting of cases is one of the ideas put forward to explain this discrepancy. The molecular developments are noteworthy, particularly the role played by single nucleotide polymorphisms (SNPs) in ACEs (ACE1 and ACE2). The virus can enter the host cell thanks to the transmembrane protein ACE2, which is a homolog of ACE1.

**Objectives:**

With a focus on the I/D genotype of ACE1 and the rs2285666 SNV of ACE2, we elucidated the prevalence of SNPs in ACE1 and ACE2 in various geographic locations. We examined the relationship between these SNPs and the global patterns of COVID-19 prevalence.

**Methods:**

66 of the 127 articles obtained using PubMed, Google Scholar, and Google directly conformed to the search terms; geographical distribution of viral infections, the prevalence of COVID-19, ACE1, ACE2, SNPs, and prevalence of the DD genotype, and rs2285666.

**Results:**

The DD genotype of ACE1 and the rs2285666 SNV of ACE2 are vital in their gene expression and contribute greatly to viral disease susceptibility, development, and severity. There was generally a high prevalence of the DD genotype in Europe and America, where COVID-19 had a more devastating effect than in Asia and Africa. The prevalence of the SNV rs2285666 varied in the following order: East Asia> South Asia >America>Europe >Africa. However, there were conflicting agreements in the association of rs2285666 with COVID-19 susceptibility and prevalence.

**Conclusion:**

The ACE1 DD genotype and COVID-19 prevalence have been positively linked in a number of studies. The ACE2 rs2285666 SNV, however, has yielded no definitive results. To determine the relationship between these SNVs and COVID-19 incidence, more research is required.

## 1. Introduction

Coronavirus disease of 2019 (COVID-19), the pandemic caused by severe acute respiratory syndrome coronavirus 2 (SARS-CoV-2), has had an appalling effect [1, 2] on humanity, with 517,648,631 cases and 6,261,708 deaths reported by the World Health Organization (WHO) as of 9th of May 2022 (WHO; https://covid19.who.int/). However, this effect in terms of infection rates, morbidity, and mortality is geographically unevenly distributed with Africa that has predominately less developed countries having one of the lowest rates while Europe and the Americas have the highest rates ([3–5]; https://covid19.who.int/). According to the WHO COVID-19 weekly report of 4th February 2022, of the 517,648,631 cases, Africa accounts for only 1.7%, while Europe and America account for the largest percentages, 42.1 and 29.8, respectively (WHO; https://covid19.who.int/). The geographical variation of COVID-19's effects substantially concurs with previous viral infections of global concern. Noteworthy, Ebola virus disease (EVD) and the Middle East Respiratory Syndrome (MERS) had geographical belonging to West Africa and the Middle East, respectively [6–9]. As a zoonotic, the geographical belonging of EVD is greatly attributed to suspected reservoirs, the wild primates, the rodents as well as the bats [8, 10], and the dromedary camels of the Arabian Peninsula for the MERS [9, 11]. Unlike EVD, cases of MERS, like COVID-19, due to human movements were reported in Europe and America, although with minimal impact compared to COVID-19 [11–13].

The most intriguing question scientists have tried to answer is why COVID-19 has had such a varied impact globally, leaning more toward the first-world regions for its greatest effects (WHO; https://covid19.who.int/). Several theories have explained this variation, with unequal screening, testing, and reporting systems globally widely attributed. However, this review will look at the contribution of the single nucleotide polymorphisms (SNPs) of cell immunological molecules, specifically the angiotensin-converting enzymes 1 and 2 (ACE1 and ACE2), which are primarily vital in cardiovascular protection [14] and through which both MERS-CoV and SARS-CoV-2 gain entry into the host cell [15].

## 2. Renin Angiotensin Aldosterone System and Angiotensin-Converting Enzyme

### 2.1. Angiotensin-Converting Enzyme 1 (ACE1)

The renin-angiotensin-aldosterone system (RAAS) is involved in blood pressure regulation. In RAAS, Renin, a vital enzyme in the conversion of angiotensinogen to angiotensin I (Ang I), is released from the juxtaglomerular cells by stimulants like decreased blood flow [16, 17]. Ang I is subsequently converted to Ang II, the main effector in RAAS. Ang II elevates blood pressure through vasoconstriction, stimulation of aldosterone secretion, and renal sodium retention [16–18]. Angiotensin-1 converting enzyme (ACE1) does the conversion of Ang I to Ang II, and noteworthy, its gene has an insertion/deletion (I/D) polymorphism on intron 16 that affects the enzymes' bioavailability. The homozygous DD and II genotypes are associated with high and low ACE1 levels, respectively [17–19]. Several studies have also associated the (I/D) polymorphism with the pathophysiology of several disorders, including cardiovascular, neurological, and recent COVID-19 [16, 17, 20–26].

#### 2.1.1. The Global Incidence of (I/D) Polymorphism and COVID-19 Cases

Several studies have looked at the effect of the ACE1 (I/D) polymorphism on the progression of COVID-19, but its contribution to disease susceptibility and hence incidence is still minimally studied. Therefore, more studies looking at the prevalence of the (I/D) polymorphism with cases of COVID-19 globally are required to achieve this. Bellone & Calvisi [27] reported a generally higher prevalence of the D allele among Europeans, with an average II, ID, and DD genotypic proportion of 0.21, 0.49, and 0.30, respectively. In total agreement, Livshits et al. [28] also reported a lower prevalence of the II genotype (18%) among Ukrainians, with an average proportion of 0.21 in general among Europeans. Interestingly, in both studies, countries like Italy and Spain with the highest COVID-19 cases and deaths, according to the weekly reports from the European Centre for Disease Prevention and Control (https://www.ecdc.europa.eu/en/cases-2019-ncov-eueea, 8, June 2022), have a more profound D allele prevalence (Table 1). Contrary to the European countries, Bellone & Calvisi [27] reported a higher I allele than the D allele, with a prevalence ratio of approximately 2 : 1 in China, which has the highest population in Asia and the lowest COVID-19 cases. This is further reinforced by Pati et al. [29] who reported a lower D allele frequency (38.43%) in China. Pati et al. [29] also reported that, with the exception of a few countries like Palestine, most of the other Asian countries that have a low percentage frequency of the D allele have had fewer COVID-19 cases and deaths compared to those that have a higher D allele frequency. The lower frequency of the D allele in Asia was also reported in admixed studies involving European and Asian countries [30, 31]. Therefore, there is a higher prevalence of the II genotype in general among Asian populations than among Europeans (Table 1). A look at the current COVID-19 cases in these two regions shows that the high prevalence of the DD genotype, to some extent, has a role to play in COVID-19 susceptibility and hence the global variation in the cases as well as deaths. Yamamoto et al. [31] reported a strong negative correlation between the II genotype with COVID-19 cases (-0.847) and associated deaths (-0.755). Statistics from studies done in Africa (Table 1: OR = 0.4, *R* = −.0245), also suggested a protective advantage of the II genotype to COVID-19 and its negative correlation with the disease.

Several studies were done on the I/D polymorphism in the USA to determine its effect on several disorders, which eventually uncovered the prevalence of the genotypes and alleles. Goodman et al. [36] & Schürks et al. [39] reported a higher DD genotype frequency (33.3 and 29%, respectively) among women, while Lindpaintner et al. [37] reported a more or less similar frequency (30.9%) of DD among men in the USA. Marson et al. [41] further corroborate this with a much higher frequency (40%) for the DD genotype and a very low II genotype frequency of 14%. Interestingly, a study that differentiated whites from African-Americans reported a significant difference in the frequency of the DD genotype, with the whites and African-Americans having 29 and 38%, respectively [38]. Therefore, most of these studies reported a significantly higher DD genotype which is associated with COVID-19 susceptibility than the II genotypes in America. This could partly explain the high number of COVID-19 cases in that region.

In Africa, the prevalence of (I/D) polymorphism has not been extensively studied, and the available literature shows that most of the studies are from the far north and southern parts of the continent. Aung et al. [33], in a global ecological study, reported a lower prevalence of the advantageous II genotype among African countries, with an average prevalence of 48 and 9.8% for the DD and II genotypes, respectively. This report concurs with other studies from South Africa and Tunisia that also reported a lower prevalence of the II genotype of 19.3 and 14%, respectively [34, 35]. Although these findings were generated using studies from a few countries—Nigeria, Tunisia, Egypt, and South Africa—which are not representative enough to give a conclusive report about Africa, these countries, except for Nigeria, have the highest COVID-19 cases on the continent (https://covid19.who.int/). A study performed in Zambia, an almost central African country, to determine the allelic and genotypic frequency of genes including ACE1 reported a very high prevalence of the protective genotype II (77.6%) compared to the DD (5%) [32]. This finding does not correlate with the number of COVID-19 cases in Zambia, which are relatively high on average (https://covid19.who.int/). Therefore, this calls for more prevalence studies on these genes of concern if conclusive reports are to be generated.

### 2.2. Angiotensin-Converting Enzyme 2 (ACE2)

ACE2, a homology of angiotensin-1 converting enzyme (ACE1), is an ectoenzyme that is transmembrane bound in the epithelial cells of numerous organs such as the heart, kidney, liver, testis, and lungs [48–50] and also in the plasma due to proteolytic shedding [50]. ACE2 plays a vital role in mitigating the cardiovascular damage of Angiotensin (Ang) II in the renin-angiotensin-aldosterone system (RAAS) by converting it to Ang (1-7) [14, 48]. Of biological importance is the role ACE2 plays in the current COVID-19 pandemic. ACE2 acts as the major receptor for SARS-CoV-2, the causative virus of COVID-19, by binding to the receptor-binding domain (RBD) of the virus and thus allowing viral entry into the cell [48, 51]. This implies that the bioavailability of ACE2 is proportional to COVID-19 susceptibility, a reason why several studies have advocated against the use of Ang II blockers and other agents that up-regulate ACE2 in the management of cardiovascular disease patients [52]. Noteworthy, several studies attribute the bioavailability of ACE2 to variants of its single nucleotide polymorphism (SNP).

#### 2.2.1. SNP in ACE2 and the Geographical SNV Stability

The scientific community has shown a great deal of interest in the ACE2 protein located on the human X-chromosome, gene XP22.2, which has 19 exons [53–55]. Numerous studies, both retrospective using previous ACE2 genetic information from databanks and “wet-laboratory” experiments, have examined the SNPs in the ACE2 gene with a common interest in ascertaining their implication on the severity and or susceptibility, especially on various cardiovascular diseases (CVD), hypertension, stroke [14, 56], and currently COVID-19 [52, 53]. This eventually emanated in the predetermination of the allelic stability of its variants. Between 6 and 16 single nucleotide variants (SNV) have been extensively analyzed to determine their association with either disease susceptibility, development, or severity. The SNV rs2285666 in the third intron that affects the expression of the gene is currently the most studied [57].

**Figure 1:**
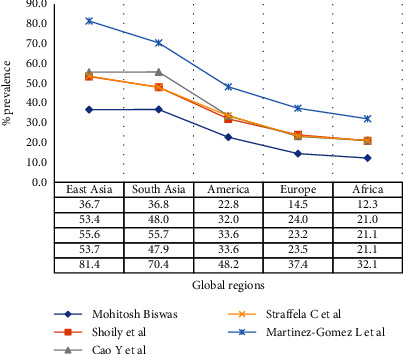
Global % prevalence of ACE2 SNV rs2285666 from different studies. Footnote: these are frequencies of the SNV rs2285666, genotypes G/A, and T/C admixed from various retrospective studies that used information from databases.

Shoily et al. [58] in trying to determine the patterns of ACE2 variants using 15 variants of disease concern in, reported a higher frequency of variants rs4830542, rs2074192, rs4240157, and rs879922 among the African population than the rest of the world. These variants, especially the rs2074192 and its G/A genotype in particular have been reported to have a protective advantage of reducing the risks of hospitalization [59]. Striking in this study, analogous to various others, was the high frequency of the variant of concern (rs2285666) among the Asian and American populations but with a rather low frequency among the Africans. A meta-analysis of this ACE2 SNV rs2285666 showed its predominance in terms of frequency (Figure 1) generally descending from East Asia, South Asia, America, Europe, and Africa [57, 58, 60–62]. These results seem to be positively correlating with the current trend of the global COVID-19 infection rate and its implications.

Several “wet-laboratory studies” have now been conducted in different geographical localities on the rs2285666 variant with oscillating agreements regarding its association with COVID-19 severity and susceptibility [59, 60, 63]. Srivastava et al. [63] reported a notably higher frequency of the variant rs2285666 among the Indian population than in other geographical areas. The results of his study showed a negative correlation, especially for the TT on the plus allele of the variant with the number of COVID-19 cases, which concurs with Alimoradi et al. [64] who reported a higher rs2285666 (G8790A) allele frequency among Iranians and particularly its GG genotype in COVID-19 Intensive Care Unit (ICU) patients than their counterparts. In Europe, similar studies on the rs2285666 variants have conflicting results. The risk of COVID-19 infection and hence the development of complications is greatly associated with this variant, especially the G-allele [53, 59] although Celik et al. [65] and Gómez et al. [66] did not find any significant association statistically.

## 3. Discussion and Conclusion

There is sufficient evidence to suggest that the ACE1 II genotype has a relative protective effect against COVID-19 compared to the DD genotype. In Asia where COVID-19 cases are less than in Europe and America, the II genotype predominates over the DD genotype (Table 1). In addition, there seems to be a trend in the increase of the II genotype prevalence from western to eastern Europe. In the case of Africa, there is very little conflicting information on the prevalence of the ACE1 I/D genotypes. Despite the low COVID-19 cases in Africa, most studies reported a high prevalence of the DD genotype, which is associated with high COVID-19 cases (Table 1). Whereas it is possible to suggest that population density had a hand in the COVID-19 cases, this argument may not explain the low numbers in China. Therefore, more studies are needed, especially on the ACE1 I/D polymorphism in Africa where COVID-19 cases were insignificant.

With the exception of South-East Asia, the global epidemiological COVID-19 cases correlated well with the prevalence of ACE2 SNV rs2285666 from the various retrospective studies (Figure 1), which would lead to the conclusion that differences in the geographical impact of COVID-19 are associated with this variant. However, most of the studies, especially those conducted as wet-laboratory studies, performed in COVID-19 high prevalence geographical areas with the intent of determining the relationship of the ACE2 SNP to disease susceptibility, development, and severity provided conflicting conclusions. Although the prevalence of the allele of concern, rs2285666, was categorized in different global geographical regions as high, moderate, and low (Figure 1), its relation to COVID-19 susceptibility is still inconclusive. Noteworthy, from COVID-19 high prevalence regions, only a few studies have associated the SNV rs2285666 with COVID-19 susceptibility. To ascertain this association better, more studies on this SNV of concern and others like rs2074192 that have a protective advantage need to be performed in high- and low-COVID-19 prevalence geographical regions of the world. These studies need to consider the COVID-19 disease variants of concern, currently Omicron, and ascertain the existence of a relationship, if any, with the SNVs.

## Figures and Tables

**Table 1:** Regional prevalence of the ACE1 genotypes and COVID-19 cases by end of June 2022.

Study	Region/countries	Genotype frequency (%)			COVID-19 cases	Risk estimate	Symmetric measures		
		DD	DI	II		OR	R	rs	95% CI
	*Africa*								
Atadzhanov et al. [32]	Zambia	5	19.2	77.6	324334				
Aung et al. [33]	Nigeria	41.8	46.2	12	256573				
	Egypt	40	55.2	4.8	514047				
Aung et al. [33]; Said et al. [34]	Tunisia	55.35	31.75	12.9	1046703				
Aung et al. [33]; Collins et al. [35]	South Africa	36.9	48.2	14.9	3986892		**-0.245**	**0.200**	**0.449-1.000**

	*America*								
Goodman et al. [36]; Lindpaintner et al. [37]; Lynch et al. [38]; Schürks et al. [39]	USA	32.04	46.76	21.22	86118591				
Alves et al. [40]; Marson et al. [41]	Brazil	36	45.85	18.15	32130316	**NA**	**NA**	**NA**	**NA**

	*Europe*								
Bellone & Calvisi [27]	All	30	49	21	22734709				
Livshits et al. [28]	Ukraine	31	51	18	5017038				
Aladag et al. [42]	Turkey	40.2	52.7	7.1	1509670				
Jacobs et al. [43]	Belgium	28.4	62.7	9	4225222				
Hubacek et al. [44]	Czech Republic	24.2	51.7	24.1	3096585				
	Spain	34	50	16	12734038				
Eleni et al. [30]; Gialeraki et al. [45]	Greece	37.7	49.3	13	3644889				
Rigoli et al. [46]; Yamamoto et al. [31]	Italy	33.4	50.0	16.6	18343422	**0.79**	**0.418**	**0.405**	**0.284-0.307**

	*Asia*								
Pati et al. [29]	China	15.3	46.3	38.4	4686464				
	Mongolia	8	50	42	928586				
	United Arab Emirates	74.5	39.6	14.2	942253				
	Nepal	12.6	43.5	43.9	979658				
	Bahrain	40.2	44.3	15.5	622261				
	Lebanon	49.1	41.0	9.9	1108965				
Ahluwalia et al. [47]; Yamamoto et al. [31]	India	23.2	41.9	34.85	43433345	**0.66**	**0.225**	**0.00**	**0.913-0.924**

OR is the odds ratio, *R* is Pearson's *R* value of association (Monte Carlo Sig. 95% CI), and rs is Spearman's rank correlation coefficient value of the linear relationship. DD, II, and DI are the homozygous deletion/deletion, insertion/insertion, and heterozygous deletion/insertion genotypes of ACE1.

## Data Availability

Data can be availed at request from the corresponding author at s.atiku@muni.ac.ug.
